# Attitudes of Implanting Physicians about Cardiac Rhythm Management Devices and Their Features

**DOI:** 10.1155/2013/247586

**Published:** 2013-12-26

**Authors:** Darryl A. Elmouchi, Nagib Chalfoun, Andre Gauri

**Affiliations:** ^1^Frederik Meijer Heart & Vascular Institute and Spectrum Health, 2900 Bradford Street NE, Grand Rapids, MI 49525, USA; ^2^Michigan State College of Human Medicine, Grand Rapids, MI 49525, USA

## Abstract

Modern cardiac rhythm management systems have become increasingly complex. The decision on which specific system to implant in a given patient often rests with the implanting physician. We conducted a multiple-choice survey to assess the opinions and preferences of cardiologists and electrophysiologists who implant and follow cardiac rhythm management systems. Reliability and battery longevity were viewed as the most important characteristics in device selection. Patient characteristics which most affected device choice were pacing indication and life expectancy. Remote technology was used in 47% of pacemaker patients, 64% of ICD patients, and 65% of CRT-D patients, with wireless (radiofrequency) remote patient monitoring associated with higher patient compliance rates (74% versus 64%, resp.). Wireless remote patient management with alerts for atrial tachyarrhythmias was felt to be important by 76% of respondents. When choosing an MR-conditional device, physicians deemed patients with prior orthopedic problems, a history of cancer, or neurological disorders to be more likely to require a future MRI. Device longevity and reliability remain the most important factors which influence device selection. Wireless remote patient monitoring with alerts is considered increasingly important when choosing a specific cardiac rhythm management system to implant.

## 1. Introduction

Modern cardiac rhythm management (CRM) devices range from single-chamber pacemakers to implantable cardioverter-defibrillators (ICDs) with cardiac resynchronization therapy (CRT). The decision of which device to implant in a given patient often rests with the implanting physician. Over the past decade, these devices have increased not only in number, but also in features offered; for that reason, many factors influence device selection. We report here on results of a survey conducted among electrophysiologists (EPs) and cardiologists aimed at determining factors that influence device selection for their patients.

A major recent breakthrough in cardiac rhythm management has been the advent of remote technology, which today is available in pacemakers as well as ICD and CRT-D systems. Remote technology today includes “remote follow-up,” in which data can be periodically downloaded from the device, either through patient interaction with a transmitter system (inductive systems) or automatically (wireless) with no patient participation. Remote follow-up allows clinics to perform periodic routine patient check-ups without the need for an in-clinic visit. Wireless systems enable the further advancement of remote technology by offering the possibility of daily monitoring, whereby the implanted device can automatically transmit messages to the clinic with no patient interaction, alerting the physician to remarkable events or conditions. Terminology describing remote technology is often unclear. For the purposes of this paper “remote follow-up” (RFU) refers to an inductive or wireless method of retrieving device information on a scheduled basis, while “remote patient monitoring” (RPM) refers to a wireless system that allows daily transmission to the clinic of specific and programmable alert messages. By this definition, all RPM is wireless, but RFU may be inductive or wireless.

RPM with daily alerts is valuable in detecting previously undiagnosed rhythm disorders, such as atrial tachycardia (AT) or atrial fibrillation (AF). AF increases the patient's risk of stroke fivefold [1]. RPM was shown in a clinical trial to reduce the risk of stroke by 9% to 18% versus conventional follow-up at 6- and 12-month follow-up intervals, respectively [2]. The ASSERT trial of 2,580 device patients found a 10.1% incidence of subclinical atrial tachyarrhythmias at three months; these episodes were associated with increased risk of stroke (HR 5.56, 95% CI, 3.78–8.17, *P* < 0.001) [3].

Another important concern for device patients is their ability to undergo imaging, should it become necessary in the future. Magnetic resonance imaging (MRI) is a valuable diagnostic tool which may be necessary for certain patient, but up until recently has been contraindicated in patients with CRM devices. Potential MRI-device interactions include threshold alterations, troponin elevations, ectopy, reed switch problems [4], and heating of the lead tip [5]. Internationally, several recent devices may now be labeled as conditionally safe for use with MRI [6]. In 2011, the first MR-conditional pacing system was approved for use in the United States.

The primary objective of this survey was to determine factors that influence cardiologists and EPs when selecting CRM devices for particular patients.

## 2. Materials and Methods

A multiple-choice telephone survey was designed to assess the opinions and preferences of EPs and cardiologists involved in the implantation and follow-up of CRM devices (pacemakers, ICDs, and CRT systems). All participants had to be in full-time active clinical practice and were blinded to the study sponsor. Owing to unique legal requirements, respondents licensed in Vermont or Massachusetts were excluded.

Survey questions could be broken down into the following broad topics:

- device selection;
- remote patient monitoring (RPM);
- MR-conditional systems.

Many device features and characteristics influence prescribing choices. Physicians were asked to rate device features on a scale of 1–5 with 5 being “extremely important” and 1 being “not at all important” in terms of the selection process. Data were analyzed by In2ition (Forest Lake, MN).

## 3. Results

At the time of the interview, 54% of respondents were in private practice and 43% were in hospital-owned practices or were employed by a hospital. See Table 1.

**Figure 1:**
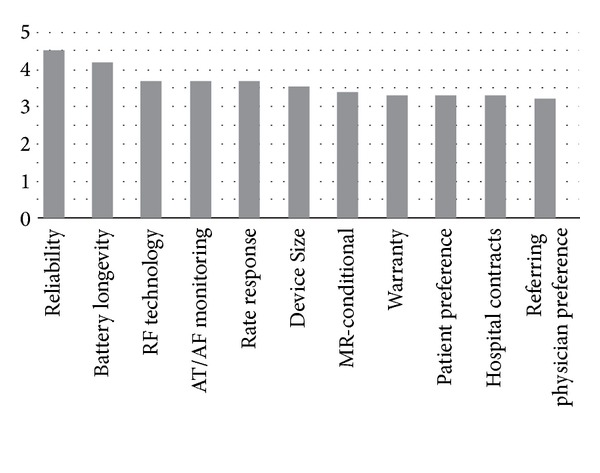
The relative importance of various CRM device features in device selection by the physician.

Survey results for device features that were examined in this study are listed in Figure 1. The most important device characteristic was reliability (rated 4.5). Battery longevity (service life) rated 4.2; wireless RPM in general, RPM with AT/AF burden monitoring, and rate-response sensors all rated 3.7. Device size rated 3.5, while MR-conditional technology rated 3.4. Patient preference, manufacturer's warranty, and hospital contracts all rated 3.3. The lowest-rated item in terms of device selection was the preference of the referring physician, evaluated at 3.2.

**Figure 2:**
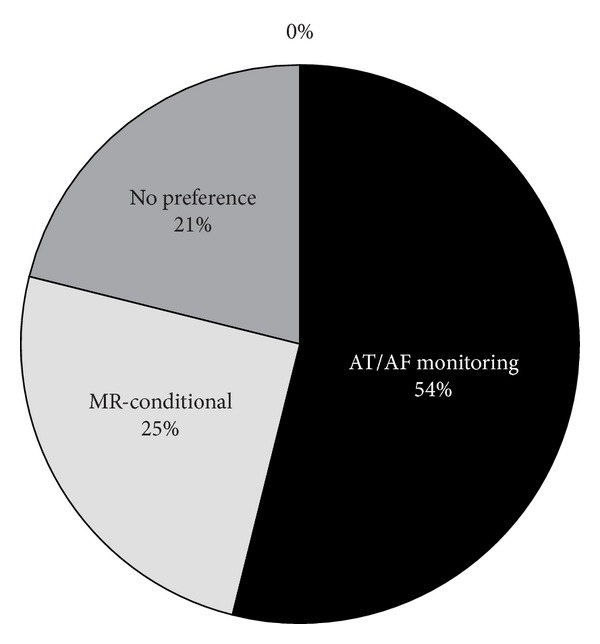
Respondents evaluated the value of AT/AF monitoring or MR-conditional devices when selecting a replacement generator.

When a pacemaker required replacement, 65% of respondents were likely to replace a pacemaker that lacked RPM capabilities with a pacemaker that offered RPM. When asked if they preferred a CRM device with RPM and daily AT/AF monitoring over an MR-conditional device, 54% of respondents preferred RPM with AT/AF monitoring versus 25% who preferred an MR-conditional device; twenty-one percent expressed no preference. See Figure 2.

**Figure 3:**
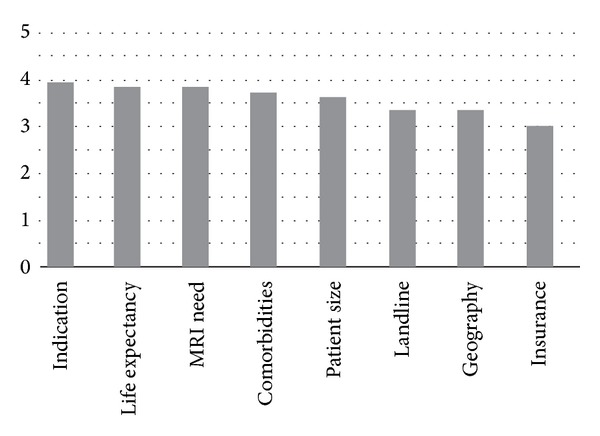
On a scale from 0 to 5, with 0 being the lowest and 5 being the highest, physicians rated patient characteristics that were important considerations for device selection.

In terms of patient characteristics considered in device selection, pacing indication and patient's life expectancy ranked highest while the patient's geographical mobility and insurance coverage ranked lowest (see Figure 3).

Remote technology in various forms is already in widespread use but is more common in defibrillator than in pacemaker systems (47% of pacemaker patients, 64% of ICD patients, and 65% of CRT-D patients). Inductive systems are more common in pacemakers (54%) than in ICDs or CRT-D systems (37% versus 34%, resp.). This suggests the eventual migration toward RPM from older forms of remote technology. See Table 2. Over 80% of respondents currently use electronic medical records in their practice. Not surprisingly, patient compliance was reported to be higher with RPM systems than with inductive systems, 74% versus 64%, respectively.

**Figure 4:**
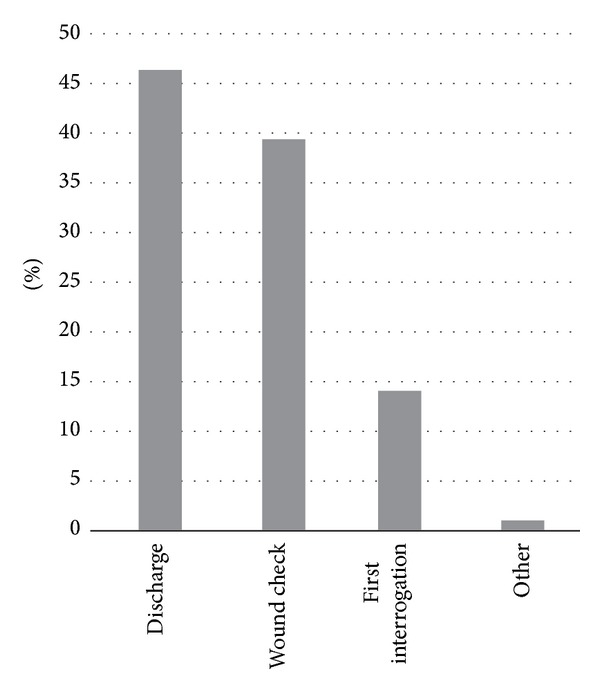
Physician preferences as to when device patients should be set up with their RPM system.

On a scale of 1 to 5 with 1 meaning “strongly disagree” and 5 meaning “strongly agree,” physicians rated the statement “sending patients home with RPM would reduce the risk of 30-day re-admissions” as 3.3 and the statement “sending patients home with RPM would reduce the risk of ER hospitalizations” as 3.5. Physicians evaluated the statement “patients are more likely to use RPM if they were set up on the system and took it home immediately following device implant” as 4.0 and the statement “patients would be more compliant if sent home with RPM immediately following device implant” as 4.0. Seventy-six percent of physicians stated that patients would be more likely to use RPM if it was set up immediately after implant. Eighty-five percent of physicians believe it would be ideal to have patients set up on an RPM system at discharge (46%) or by wound check (39%). Fourteen percent of physicians said the ideal time to set up the patient with the RPM system was at first interrogation of the device. See Figure 4.

**Figure 5:**
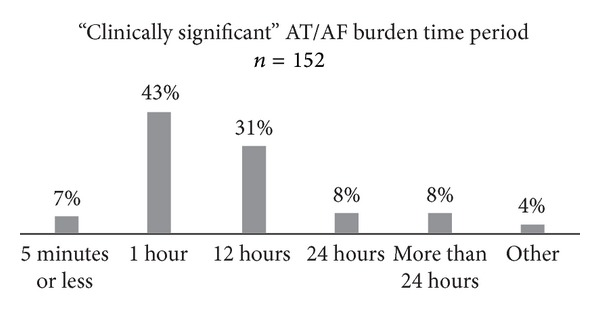
Amount of AT/AF burden time physicians considered to have clinical significance for the patient.

RPM systems with daily alerts can help document remarkable arrhythmic episodes, such as AT/AF. In device patients without RPM, a quarter of physicians reported they found evidence of previously undiagnosed AF during a device interrogation. In device patients with RPM, AT/AF alerts were able to report previously undiagnosed episodes of silent AF in 27% of all patients. While ICDs and CRT-Ds often offer RPM with daily AT/AF alerts, this feature is less common in pacemakers. Seventy-six percent of physicians said it was “very important” or “important” for pacemakers to offer RPM with AT/AF alerts. Although physicians found AT/AF alerts important for pacemakers, they also said that such alerts (in all devices) were useful to them only about 45% of the time. Physicians stated that RPM with AT/AF alerts was used 50% of the time in order to reduce the patient's risk of stroke. Seventy-five percent of physicians reported being “very concerned” or “concerned” about clinically silent AF. When asked what was felt to be a clinically significant AT/AF burden, 7% of respondents said five minutes or less while 43% stated an hour. See Figure 5.

Notification speeds (from device to clinic) are not yet standardized in the industry. Notifications that an AT/AF episode exceeded the programmed duration were desired by 22% of respondents within 24 hours and 52% wanted lead impedance notification within 24 hours. See Table 3.

Two-thirds of respondents reported that they have had device patients rehospitalized for an AF-related event within three months after implant. Based on timeframe windows, rates were similar by device types. Further, 67% of respondents reported having had device patients readmitted to the hospital within 90 days of implant for an AF-related event. See Table 4.

Respondents stated that 14% of their pacemaker patients have, at some point, needed an MRI. Physicians in practice for more than 10 years were significantly more likely to have seen pacemaker patients require MRIs than those in practice for 10 years or fewer (17% versus 10%, resp., *P* < 0.05). The patients who are most likely to receive an MR-conditional device are those with orthopedic problems (4.2 rating on a 1–5 scale with 1 being “not at all likely” and 5 being “very likely”), a history of cancer (4.1), neurological issues (4.0), and a history of prior MRIs for nonchronic comorbid conditions (3.8) and those aged 41 to 60 (3.7) or aged ≤40 years (3.7). Least likely to get an MR-conditional system are those aged 81 or older (3.2), CRT-D patients (3.2), and ICD patients (3.1). Of physicians who have implanted an MR-conditional device, 43% have had a patient undergo an MRI scan.

In this survey 64% of respondents had experience implanting an MR-conditional device.

## 4. Discussion

The landscape of implantable cardiac devices has changed rapidly in the past few years. Broader indications for ICD and CRT implantation [7–12] as well as an aging population have expanded and will continue to expand the use of CRM systems. Far from the early single-chamber, fixed-rate pacemakers, devices today not only treat, but also help diagnose disease. With increased device complexity, there is a concomitant increased concern about device and/or lead malfunction. In light of recent device recalls and increasingly sophisticated technologies, the Heart Rhythm Society has called for expanded use of RPM in the interest of patient safety [13].

This survey sought to understand factors which influence device selection and the attitudes held by implanting physicians related to new device technology. A broad mix of implanting cardiologists and EPs were surveyed, representing private practices, hospital-owned or affiliated groups, and academicians. This was a group that had been in practice for over 11 years on average and was primarily responsible for device selection at their institutions.

Perceived device reliability was the single most important factor in device selection. Following this, battery life was a close second [14, 15]. In the REPLACE study, pacemaker and ICD patients were followed for six months after generator change or system upgrade. Major complications were reported in 4.0% to 15.3% of patients in this trial. Since complication rates increase markedly at the point of generator change, this emphasis on device reliability and longevity is well founded.

Interestingly, next on the list for factors influencing device selection were rate-response sensors and RPM with AT/AF alerts. While rate response has been available since the 1990s, wireless technology allowing the use of RPM is a feature which was minimally available even a few years ago. The sudden rise in importance of RPM allowing the use of AT/AF and other alerts speaks to the apparent clinical value of such features. These new features have quickly gained widespread acceptance in routine clinical practice because they can be quickly and readily deployed, provide relevant clinical data, and align with modern clinical goals of enhancing patient safety without increasing clinical workloads.

The use of RPM with daily alerts was felt to offer many important benefits for both patients and device clinics. A large number of physicians had cared for patients who had been readmitted to the hospital or an emergency room because of an AF-related event within 30 to 90 days after device implantation. The ability to reliably and expeditiously detect AT/AF may help minimize the risk of a thromboembolic complication. It is unclear how much AT/AF burden on RPM is necessary to represent an increased risk of complications. However, in this survey, 43% of respondents felt that even an hour of high-rate atrial activity was important enough to help guide clinical decision making. Nearly real-time data such as this could only be identified via RPM. Half of the surveyed physicians felt that RPM with alerts could minimize stroke risk among device patients. As our healthcare system shifts more towards prevention of illness and avoidance of rehospitalizations, remote and rapid diagnosis of AF will likely grow in importance. Further studies are warranted to determine if early remote diagnosis may lead to fewer and less prolonged hospitalizations for patients with AF.

Compliance is always an important factor in good clinical care. Respondents felt that patient compliance using RPM with daily alerts would be more likely if the system was set up immediately after implant. Currently, most patients receive a remote monitor weeks to months after implant. Not surprisingly, patient compliance is suboptimal. RPM systems may also be given to patients or their families at first interrogation, at wound check, or at discharge. In our clinic, we follow over 6,500 implantable cardiac rhythm management device patients—5,000 of whom are followed remotely. Since early 2011, we instituted a program where all patients with qualifying devices receive their RPM system immediately upon hospital discharge rather than downstream or by mail. This has increased RPM usage significantly within our clinic and appears to be in line with the attitudes of the implanting physicians in our survey. In addition, survey respondents felt that compliance with home monitoring was increased when using RPM devices. This is likely due to the automation and lack of patient effort required when using RPM compared to RFU.

MR-conditional devices have been recently released to the global marketplace, and this survey helps to better understand why an MR-conditional device might be chosen for a given patient. Not unexpectedly, there are many factors which influence the selection of an MR-conditional device for a given patient. The patient's life expectancy is a consideration and, equally as important, his or her likelihood of needing an MRI in the future. In this light, patients with prior orthopedic or neurologic problems or a history of cancer were considered to be more likely candidates for an MR-conditional device, probably because physicians assumed they were the most likely to need an MRI in the future.

The wealth of new features and technologies available in currently marketed CRM devices offers clinicians and their patients unprecedented diagnostic and therapeutic options as well as important advanced device characteristics, such as extended longevity and compact device size. While clinicians today have more choices than before, there is still no perfect device. Selecting the best-possible device features and characteristics for an individual patient can still be a balancing act, where the physician must determine which features will be of great value to the patient now and in the future. Choosing the most relevant features for a patient today can be a bit of a guessing game in that modern devices can last five to ten years or longer. Determining the appropriate device for an individual patient can be a trade-off: MR-conditional system or RPM device? Small device size and light weight versus extended longevity? As further studies demonstrate the potential benefits of new device technologies, deciding between them may become even more difficult. This survey helps to clarify some of the factors that currently influence how implanting physicians evaluate devices for their patients.

## 5. Limitations

All surveys have limitations in terms of selection of respondents. Questions and multiple-choice answers may not have always permitted respondents to qualify answers as thoroughly as an interview format. Furthermore, this survey asked respondents to quantify their opinions but did not evaluate their actual practice. It is possible that the opinions expressed in this survey may not accurately mirror clinical practice.

## 6. Conclusion

Our survey helps shed light on factors influencing physicians' choice of cardiac implantable devices. Device reliability and battery longevity are the most important factors when considering which device to implant. The use of wireless RPM with daily alerts has increased dramatically as has the perceived importance of this groundbreaking technology to implanting physicians. The use of RPM is considered important in order to clinically manage patients and their CRM systems. MR-conditional devices have reached the marketplace and their use will likely increase in coming years. As device technology evolves, it is likely that implanting physicians will have more choices—and possibly fewer trade-offs—to make in the future.

## Figures and Tables

**Table 1:** Respondent demographics.

	Respondents
*n*	156
Years after residency or fellowship	12.0
Devices implanted per month	32
Pacemakers implanted per month	13
ICDs implanted per month	9
CRT-D systems implanted per month	6
CRT-P systems implanted per month	3
Academic affiliation	35%
“Completely responsible” for device selection	72%
“Mostly responsible” for device selection	23%
Use of electronic records	85%

**Table 2:** The use of inductive versus (wireless) RF technology in remote systems by device type.

	Pacemaker	ICD	CRT-D
Inductive remote systems	54%	37%	34%
Wireless (RF) remote systems	46%	64%	66%

**Table 3:** Preferred timeframes for RPM alert notifications.

	<24 hours	24–48 hours	Next in-office visit	Not applicable
ERI	23%	52%	22%	3%
Lead impedance	52%	36%	11%	1%
AT/AF episode duration	22%	51%	25%	2%
AT/AF burden	22%	51%	25%	2%
High-rate V episodes	22%	51%	25%	2%

AT/AF: atrial tachyarrhythmia/atrial fibrillation.

ERI: elective replacement indicator.

V: ventricular.

**Table 4:** Respondents who have ever had device patient(s) hospitalized for an AF-related event following implantation, by device type.

	0–30 days post-implant	31–60 days post-implant	61–90 days post-implant
Pacemaker	35%	30%	67%
ICD	36%	42%	66%
CRT-D	34%	32%	77%

## Abbreviations

AF: Atrial fibrillation
AT/AF: Atrial fibrillation/atrial tachyarrhythmia
CRM: Cardiac rhythm management
CRT: Cardiac resynchronization therapy
CRT-D: Cardiac resynchronization therapy device with defibrillator
CRT-P: Cardiac resynchronization therapy pacemaker
EP: Electrophysiologist
ICD: Implantable cardioverter-defibrillator
MR-Conditional: Magnetic resonance conditional
MRI: Magnetic resonance imaging
RF: Radiofrequency
RFU: Remote follow-up
RPM: Remote patient management.
